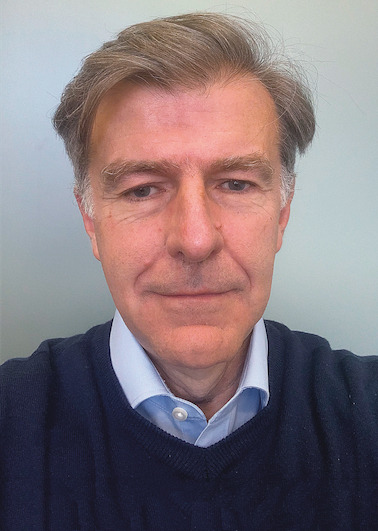# Imaging Amyloid‐β Membrane Interactions: Ion‐Channel Pores and Lipid‐Bilayer Permeability in Alzheimer's Disease

**DOI:** 10.1002/anie.202215785

**Published:** 2023-03-30

**Authors:** John H. Viles

**Affiliations:** ^1^ Department of Biochemistry, SBBS, Queen Mary University of London UK

**Keywords:** Amyloid, Annular Oligomers, Electron Microscopy, Protofibrils, Structure

## Abstract

The accumulation of the amyloid‐β peptides (Aβ) is central to the development of Alzheimer's disease. The mechanism by which Aβ triggers a cascade of events that leads to dementia is a topic of intense investigation. Aβ self‐associates into a series of complex assemblies with different structural and biophysical properties. It is the interaction of these oligomeric, protofibril and fibrillar assemblies with lipid membranes, or with membrane receptors, that results in membrane permeability and loss of cellular homeostasis, a key event in Alzheimer's disease pathology. Aβ can have an array of impacts on lipid membranes, reports have included: a carpeting effect; a detergent effect; and Aβ ion‐channel pore formation. Recent advances imaging these interactions are providing a clearer picture of Aβ induced membrane disruption. Understanding the relationship between different Aβ structures and membrane permeability will inform therapeutics targeting Aβ cytotoxicity.

## Graphical abstract

Self‐association of Amyloid‐β (Aβ) into oligomeric, protofibril, annular and fibril assemblies is intimately linked with neuronal toxicity and Alzheimer's disease. Aβ can have a range of impacts on the lipid membrane including a carpeting effect and ion‐channel pore formation. Imaging these interactions with the lipid bilayer is providing a clearer picture of Aβ‐induced membrane permeability, which leads to loss of cellular homeostasis.

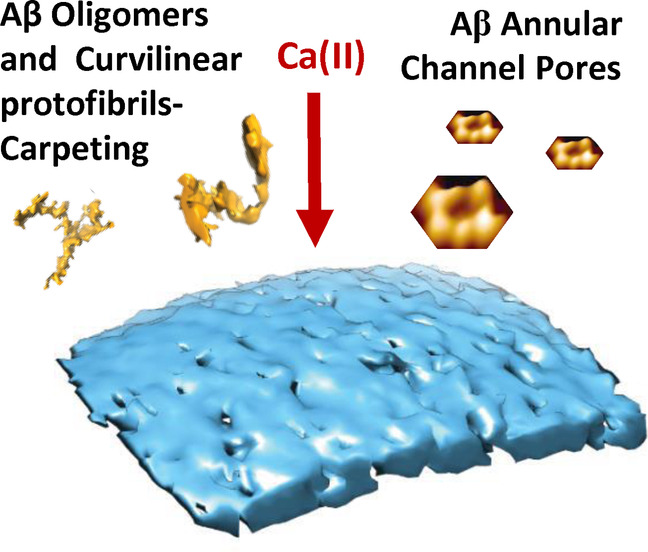

## 1 Alzheimer's Disease: An Overview

Alzheimer's disease (AD) is a fatal neurodegenerative disorder responsible for ca. 70 % of dementia cases worldwide. Life expectancy across the world is projected to improve year‐on‐year, consequently it is estimated that the number of people living with dementia will rise from 50 million today, to 132 million by 2050. Public health resources are set to be stretched even further, to cope with this all too common, progressive, debilitating and ultimately fatal disease.^[1]^

### 1.1 Aβ Oligomers and the Amyloid Cascade

A key feature of the molecular processes surrounding AD is the accumulation of a short hydrophobic peptide, amyloid‐β (Aβ).^[2]^ This peptide can have a variable N‐ and C‐terminus but is typically 40 or 42 amino acids in length.^[3]^ A key hallmark of AD, is Aβ aggregation and the formation of extracellular senile plaques within the brain interstitium and vasculature.^[4]^

Aβ is an endogenous peptide that is cleaved from a larger transmembrane amyloid precursor protein (APP) by the action of the β‐secretase and γ‐secretase complex, which includes the presenilins, PS1 and PS2.^[5]^ There is a large body of evidence to indicate that over production, increased aggregation, or reduced clearance of Aβ, is the key event in the pathology of AD.^[6]^ A clear direct link between Aβ and AD is apparent for those with inherited early‐onset AD. Over 25 mutations in APP and 100 in PS1, are associated with incidence of familial AD.[^5a^, ^5c^] While for the more common late‐onset AD, those with the ϵ4 allele of apolipoprotein (APOE‐ϵ4) have an increased risk of developing AD. This ϵ4 allele is associated with impaired Aβ clearance.^[7]^ These observations have led to the amyloid cascade hypothesis which describes the aggregation of Aβ into both small oligomers and larger fibril assemblies, which leads to a loss of cellular homeostasis. A cascade of molecular and cellular events ensues, including: dendritic spine shrinkage; loss of synaptic connection^[8]^ and loss of long‐term potentiation (LTP);^[9]^ as well as mitochondrial and oxidative stress.^[10]^ This is followed by an altered balance of kinase and phosphatase activity which results in hyper‐phosphorylation of the microtubule associated Tau protein,^[11]^ and the formation of intra‐cellular neurofibril tangles of Tau, which finally culminates in cell death and dementia.^[6]^

A critical early step in disease progression is the misfolding and self‐assembly of monomeric Aβ into fibrillar aggregates, via a range of metastable oligomeric and protofibrillar intermediates.^[12]^ These assemblies have different biophysical and synapto‐toxic properties, and it is the oligomers of Aβ_42_, ranging from 9–200 kDa, that have been shown to be the most neurotoxic.[^9^, ^13^] Although Aβ_40_ is the most abundant isoform,^[14]^ Aβ_42_ has been shown to be the principle cause of neurotoxicity^[15]^ and familial mutations which result in early‐onset AD, are linked to an increase in the ratio of Aβ_42_ to Aβ_40_.^[16]^

### 1.2 An Overview of Aβ Membrane Disruption

The ability for Aβ to bind and disrupt cellular membrane integrity has received much interest as a mechanism whereby Aβ can impede cellular homeostasis,^[17]^ this triggers a cascade of events that ultimately leads to neuronal cell death. Ca^2+^ has the largest ion gradient across the cellular membrane; five orders of magnitude. Thus, cellular membrane permeability results in Ca^2+^ influxes. Elevated cytoplasmic Ca^2+^ leads to mitochondrial oxidative stress, excitoxicity and toxic cell injury, which is observed in AD pathology.^[18]^

Aβ membrane interactions can occur via the neuronal plasma membrane but also subcellular compartments, such as those of the mitochondria.[^10a^, ^19^] Aβ has high affinity for the phospholipid cardiolipin, found in mitochondrial membranes.^[20]^

There are various explanations for the mechanism by which Aβ can compromise membrane integrity, these include: ***(i) Ion Channel Formation***: Aβ can form ion‐channel pores which span reconstituted artificial lipid membranes.^[21]^ More recently, it has been shown that only Aβ_42_ oligomers are able to insert into cellular membranes and form large single ion‐channel pores, with conductance measurements indicating an internal diameter of *ca* 1.7, 2.1 or 2.4 nm.^[22]^ ***(ii) Carpeting Effect***: A more wide‐spread carpeting and insertion into the upper‐leaflet of the membrane by Aβ oligomers has been imaged by cryoET.^[23]^ This can cause a general increase in membrane conductance due to lateral spreading of phospholipid head‐groups, which is sometimes described as membrane thinning.^[24]^ ***(iii) Detergent Effect***: A detergent‐like lipid extraction by Aβ oligomers, from supported lipid bilayers, has been imaged using atomic force microscopy (AFM),^[25]^ during this extraction lipids can become bound to the growing fibrils.^[26]^ Indeed, Aβ plaques also have a high lipid content.^[27]^ **(iv)** ***Receptor Mediated Effect***: There are a number of membrane associated proteins, such as the cellular prion protein (PrP^C^)^[28]^ and glutamate receptors such as N‐methyl‐D‐aspartate receptor (NMDAR) that Aβ can bind to and as a consequence may also perturb cellular homeostasis.[^17c^, ^29^] **(v) Oxidative damage**: Once cell homeostasis is compromised oxidative stress will generate reactive oxygen species (ROS), such as the hydroxyl‐radical, this causes lipid peroxidation and so compromises membrane integrity further.^[10b]^

Many of these effects on lipid membranes have been observed for other amyloid forming proteins, such as alpha‐synuclein^[30]^ and amylin (IAPP‐ islet amyloid polypeptide)^[31]^ and others.[^17b^, ^32^, ^33^] Furthermore, some of the impacts on the membrane draw parallels with the toxic action described for anti‐microbial peptides.^[17b, 34]^ Antimicrobial peptides will insert into lipid membranes and form pores across the membrane in a similar manor to Aβ.^[35]^ Indeed, it is suggested that Aβ itself has antimicrobial properties.[^34b^, ^36^]

### 1.3 Aim of Review

The complicated picture of Aβ membrane interactions reflects the multitude of effects Aβ is capable of exerting on the membrane. The range of observations may also be due to different techniques and membrane models employed, which have resulted in various aspects of the Aβ membrane interactions being emphasized. This review aims to bring these differing accounts together, to reconcile conflicting observations and to provide a more holistic view of Aβ‐membrane interactions.

## 2 Aβ Assembly and Structure

**Figure 1:**
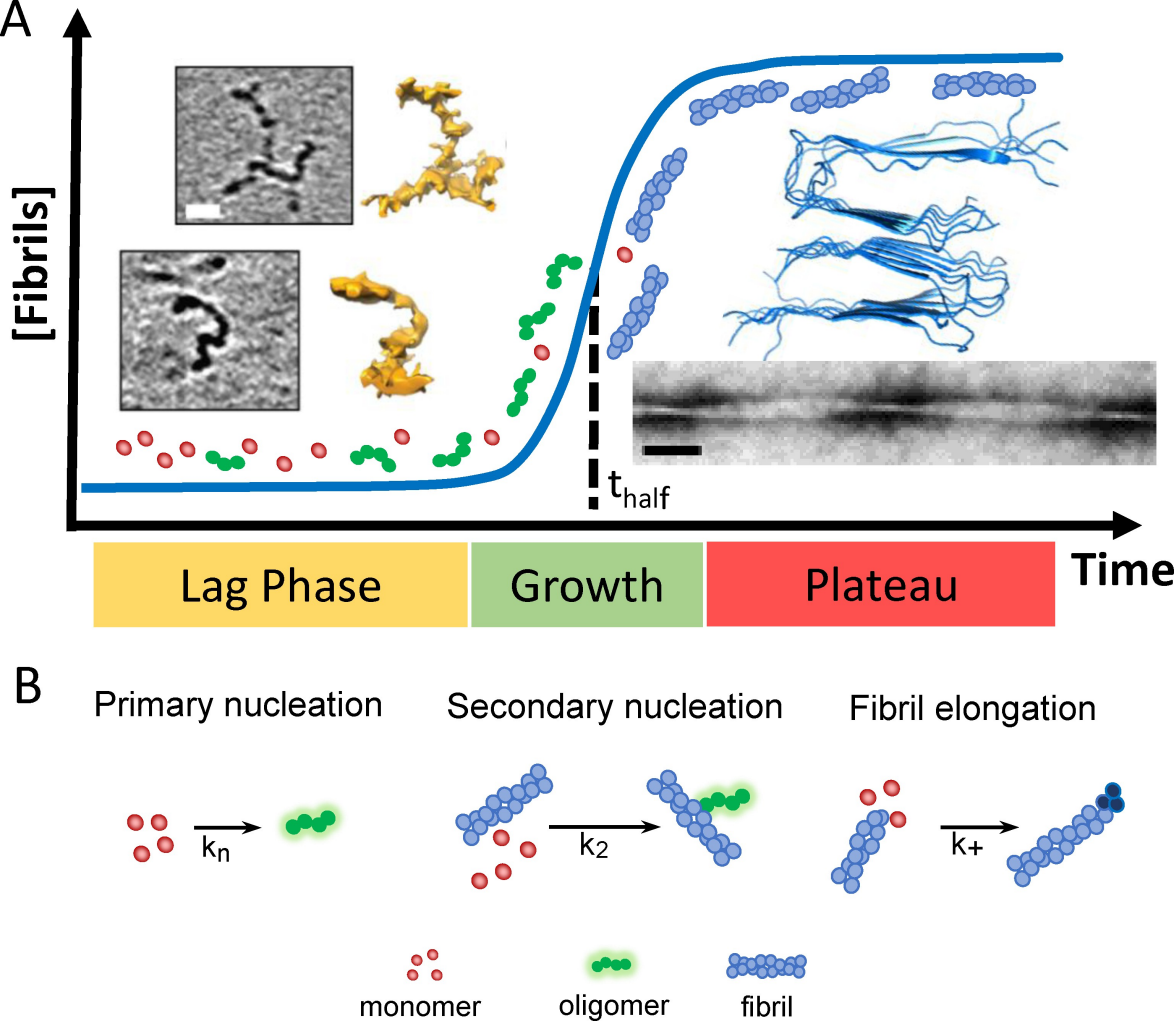
Aβ assembly and structure. (A) Sigmodal fibril growth curve, lag, growth and plateau phases. At end of the lag‐phase oligomers and curvilinear protofibrils (and monomer) dominate, while at the plateau fibrils dominate. CryoET images of Aβ_42_ curvilinear protofibril approximately 2.7 nm diameter and up to 40 nm in length, scale bar: 10 nm.^[23]^ Amyloid fibrils for Aβ_40_, PDB:2LMO^[50a]^ and TEM image, scalebar 50 nm. (B) Key microscopic process of fibril formation with rate constants for primary k_n_; secondary k_2_ and elongation k_+_ rates.[^39^, ^45^]

Different Aβ structures exhibit very different impacts on lipid membranes. A clear description of Aβ self‐assembly from monomeric peptide through to amyloid fibrils will inform our understanding of Aβ‐membrane interactions and assist rational drug design.^[37]^ A well‐studied aspect of the molecular events surrounding AD is the misfolding and assembly of monomeric Aβ peptide into amyloid fibrils.^[12b]^ The kinetics of this process is described as a nucleation polymerization reaction.^[38]^ In vitro, the kinetic growth curves of amyloid fibril formation, over time, have a sigmodal appearance, Figure 1A, with a lag‐phase in which nucleating oligomers are formed, followed by rapid formation and extension of fibrils, known as the elongation phase.^[39]^ Finally, fibril formation plateaus as monomer concentrations are depleted and an equilibrium phase is reached. Fibril formation is typically monitored using a fibril specific fluorescent dye, such as Thioflavin‐T (ThT).^[40]^ Rate constants for individual microscopic molecular processes can be obtained by globally fitting macroscopic kinetic behaviour, at multiple Aβ concentrations.^[41]^ Rate constants for: the primary nucleation; secondary fibril surface catalysed nucleation; and the elongation rate on the ends of growing fibrils can be obtained, Figure 1B.[^41^, ^42^] A particular property of the nucleation polymerization reaction is the ability for fibril growth to be accelerated by self‐ and occasionally cross‐seeding nucleation.^[43]^ A reduction in pH within micro‐environments, such as those found in the endosome and lysosome, will accelerate oligomer and fibril formation,^[44]^ this is caused by an increase in the rate of primary nucleation as Aβ becomes more neutrally charged.^[45]^ In addition, there are various factors in vivo that impact assembly into fibrils, including the presence of metal ions,^[46]^ protein binding partners^[47]^ and lipid membranes (see Section **7**).^[48]^

The assembly of Aβ leads to a mixture of Aβ oligomeric, curvilinear protofibrils and annular oligomeric structures, while at equilibrium long unbranched amyloid fibrils dominate, Figure 1A. Mature fibrils can be many microns in length and range between 6–20 nm in diameter depending on the polymorph.^[49]^ Various fibril structures of Aβ_42_ and Aβ_40_ have been reported both in vitro and ex vivo using ssNMR and cryoEM.^[50]^ Fibril structures involve a cross‐β arrangement where β‐sheets, formed by intermolecular hydrogen‐bonding, are orthogonal to the fibril long‐axis.^[51]^ The topology of Aβ_40_ and Aβ_42_ are quite different with ‘U′ and ‘S′ shaped arrangements of β‐strands respectively.[^50a^, ^50b^, ^50c^, ^52^] The lateral surface of the fibrils can act as a template for secondary nucleation^[42b]^ which has different properties compared to the ends of elongating fibrils, that exhibit exposed hydrophobic side‐chains.^[53]^

Structural details of Aβ prefibrillar assemblies are more of a challenge as these structures are meta‐stable and heterogeneous.^[54]^ Studies so far have included: NMR;[^12a^, ^54a^, ^54d^, ^55^] AFM^[56]^ and cryoET.^[23]^ In addition to Aβ‐dimers, the smallest of oligomers may be just 3–6 Aβ molecules in size, roughly spherical in shape and 2–3 nm in diameter, while curvy‐linear protofibrils are elongated. CryoET indicates these curvilinear protofibrils have quite a consistent cross‐section of 2.7±0.4 nm.^[23]^ Similarly, AFM reports curvilinear protofibrils with heights of 3 nm.[^56a^, ^56c^, ^56d^] ssNMR has described a Aβ β‐barrel hexamer structure, 3 by 3 nm, as the building blocks of curvilinear protofibrils.^[54d]^ The length of curvilinear protofibrils are variable, typically between 10 and 20 nm, but the majority do not typically exceed 40 nm. The variability in the curvature of these structures is particularly apparent in the 3D cryoET images represented as single‐threshold surfaces, see Figure 1A.^[23]^

Determining oligomeric structures embedded within the membrane are of particular interest, described in the following sections. An Aβ octamer forms an anti‐parallel β‐sandwich structure within a membrane,[^57^, ^58^] while annular oligomers have been imaged by AFM embedded within reconstituted membrane.[^32^, ^59^]

## 3 Model Membranes and Lipid Bilayer Composition

**Figure 2:**
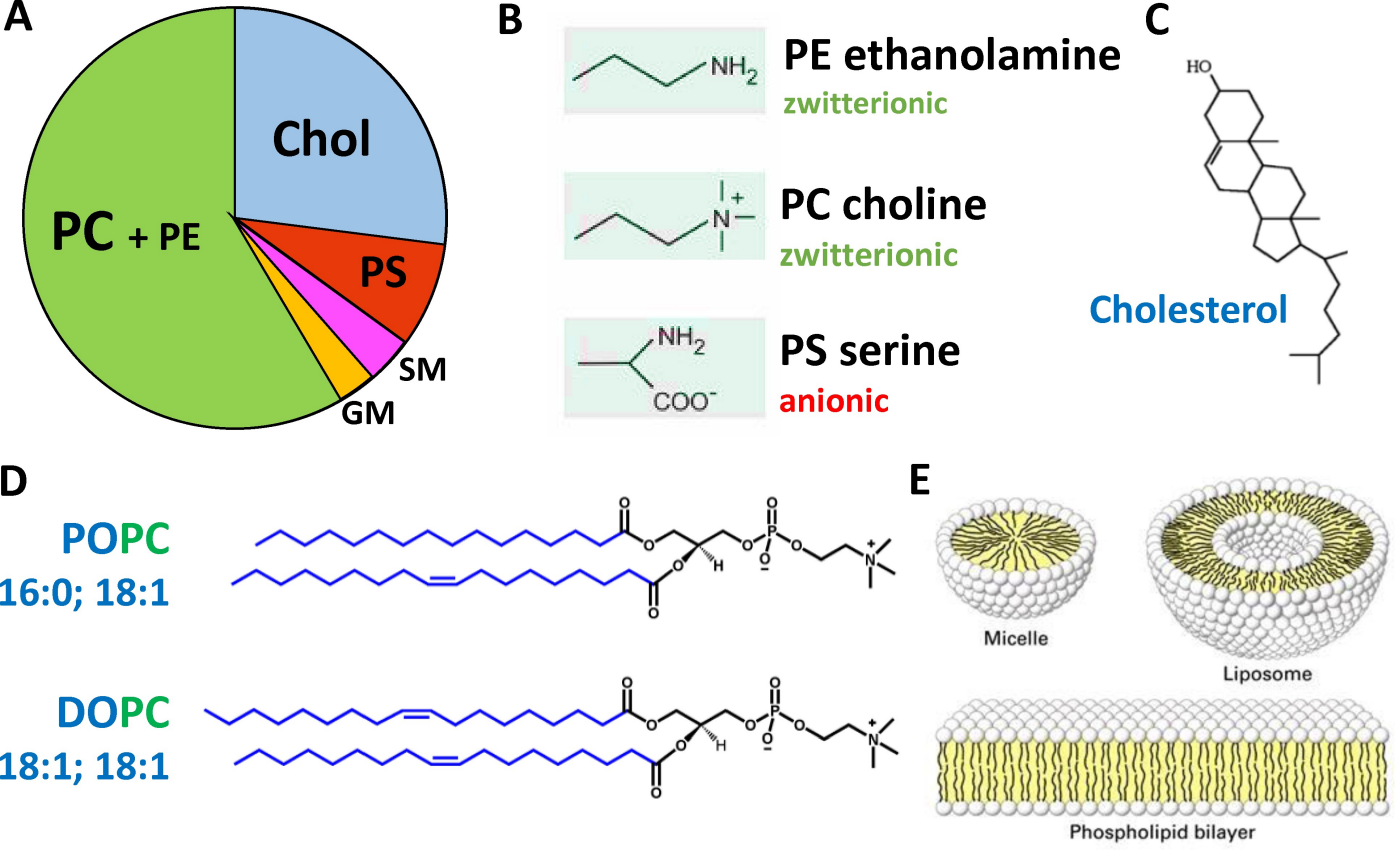
Composition and structures of the lipid bilayer. (A) Chart showing approximated proportions of key lipid components of neuronal membranes by weight.^[62]^ PC=Phosphatidylcholine; PE=phosphatidylethanolamine; PS=phosphatidylserine; GM=ganglioside; SM=sphingomyelin. (B) Some key phospholipid headgroups. (C) Cholesterol structure. (D) Two of many acyl chain combinations, 16:0–18 : 1 PS (POPS) *1‐palmitoyl‐2‐oleoyl‐sn‐glycero‐3‐phospho‐L‐serine; 18 : 1–18 : 1* (DOPC) 1,2‐Dioleoyl‐sn‐glycero‐3‐phosphocholine. (E) Cartoon of membrane structures.

Studies of Aβ membrane interactions are often most readily performed on synthetic lipid bilayers. This approach enables specific Aβ‐membrane interactions to be probed by altering the lipid‐bilayer composition. Neuronal membranes contain a complex mixture of different phospholipids which can have an array of charged head‐groups, acyl‐chain lengths and saturation.^[60]^ Cholesterol and transmembrane proteins are also major components of the bilayer. In addition, there are differences in composition of the inner and outer‐leaflet, as well as the plasma and mitochondrial membrane. Further complexity comes from the presence of micro‐domains within the bilayer, with for example elevated cholesterol, sphingolipids and ganglioside.^[61]^ The neuronal plasma membrane lipid composition contains typically 19 % by weight of cholesterol (non‐esterified). Phospholipids can be 62 % zwitterionic (mainly PC=Phosphatidylcholine and PE=phosphatidylethanolamine) and anionic 12 % (mainly PS=phosphatidylserine) by weight, Figure 2.^[62]^ However, the outer‐leaflet contains most of the cholesterol and depleted phosphatidylserine (PS), thus a reasonable first approximation of the membrane composition of the neuronal outer‐leaflet is cholesterol‐30 % and phosphatidylcholine (PC)‐70 %, by weight. Small amounts of ganglioside, such as GM1 (1 % by weight) and sphingomyelin (SM) (3 % by weight) are also important lipid components of the neuronal membrane.[^61^, ^63^] These phospholipids can be obtained from animal sources (such as egg or rat) and contain mixtures of different sidechains, which are a good approximation to the mixture of acyl chains found in neurons. Alternatively, a lipid mixture derived from total brain lipid extract can be used, with additional PC added to mimic the outer neuronal membrane. Specific phospholipids can also be used, such as POPC and POPS (PO=16:0+18 : 1; length:saturation) which are the most abundant in neurons. In addition, curvatures of the bilayer can be modelled using different sizes of liposomes (small (SUV), large (LUV), giant (GUV) unilamellar vesicles). Other membrane models such as supported bilayers, linear bilayers, mono‐layers, membrane nano‐discs and micelles are also used. The benefits and drawbacks of each system have been reviewed.[^17d^, ^64^] Cellular and synthetic lipid membrane models with different compositions, curvatures and properties may interact with Aβ quite differently. This can lead to a range of different observations which are discussed in detail in the following sections.

## 4 Visualising Aβ—Membrane Interactions

A good deal of effort has been devoted to imaging the interactions of Aβ assemblies with lipid‐bilayers as these can disrupt the integrity of the membrane. These include studies using predominantly Atomic Force Microscopy (AFM)[^25^, ^65^] and negatively‐stained samples for transmission electron microscopy (TEM).[^25^, ^46a^] These imaging techniques can reveal nanoscale details of the membrane‐amyloid interaction but can be prone to artefacts. More recently, with improvements in direct detection cameras, cryo‐electron tomography (cryo‐ET) has become a powerful imaging technique and has been used to image Aβ interactions with lipid bilayers from vesicles without the need for heavy metal staining or the use of a bilayer support.^[23]^

### 4.1 CryoET Imaging of Liposomes

**Figure 3:**
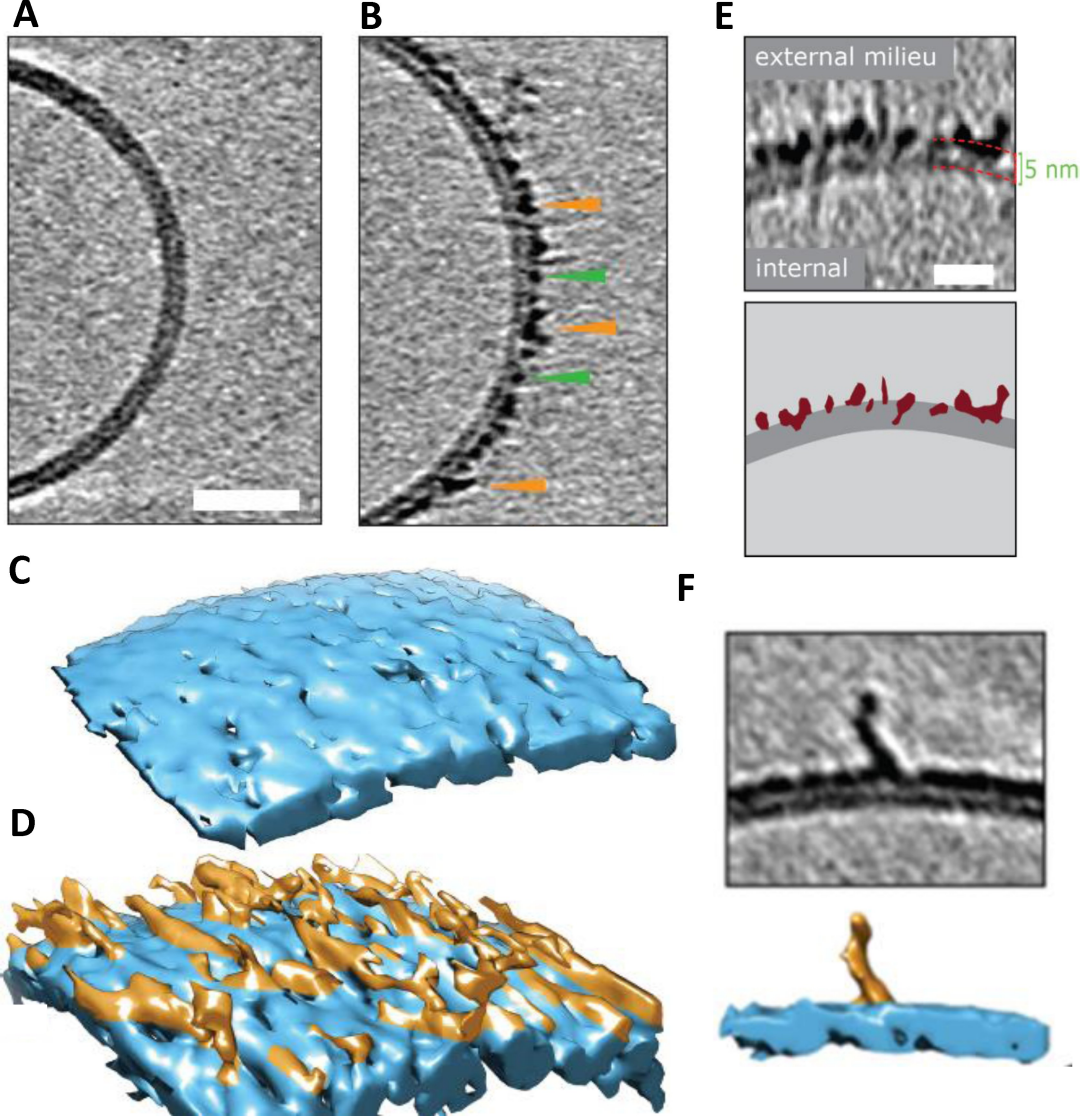
Cryo‐electron tomographic images of Aβ42 curvilinear protofibrils and oligomers on lipid vesicles. (A) Large unilamellar vesicle with Aβ_42_ monomer, indistinguishable from vesicles with no Aβ. (B) Lag‐phase Aβ_42_ oligomers/protofibrils decorate the outer surface of the bilayer. Tomographic slices are 7.6 nm thick, green and orange arrowheads highlight oligomers and curvilinear protofibrils, respectively. Scale bar: 25 nm. (C–D) 3D single threshold rendered surface, image shows lipid bilayer, 5 nm thick (blue) in the absence and presence of Aβ oligomers/protofibrils carpeting the membrane. (E) The heightened density indicates Aβ oligomer are also inserted within the membrane, Aβ protofibrils and oligomers (burgundy) inserting into the lipid bilayer (dark grey), scale bar: 10 nm. (F) 3D single threshold rendered surface; image shows curvilinear protofibrils orthogonal to membrane. Adapted from [23].

CryoET can resolve unique structures in a near native state, in three dimensions and in the macromolecular resolution range. It is particularly well suited to investigate protein‐membrane systems.^[66]^ CryoET has recently been used to image the impact of Aβ on lipid vesicles.^[23]^ Different Aβ assembly forms have been incubated with large unilamellar lipid vesicles (LUVs) containing; PC, Cholesterol and GM1 (68 : 30 : 2 by weight). The 3D images highlight the impact different Aβ assembly forms have on lipid bilayers. Chromatographically purified monomeric Aβ_42_ has no detectable impact on the appearance of the lipid bilayer, according to cryoET, Figure 3A. In contrast, Aβ_42_ oligomers and curvilinear protofibrils (taken at the end of the lag‐phase of amyloid assembly) bind extensively to the lipid vesicles, embedding and carpeting the upper‐leaflet of the bilayer, Figure 3B. The rendered 3D surface highlights the carpeting of membranes by the Aβ_42_ oligomeric and curvilinear assemblies, Figure 3C,D.^[23]^ The heightened contrast‐density of Aβ oligomers and protofibrils enables imaging of the Aβ assemblies penetrating and inserting into the upper‐leaflet of the bilayer, Figure 3E. This will cause lateral spreading of the phospholipid head groups, with the curvilinear protofibrils orientated orthogonally to the membrane surface, Figure 3F. The Aβ oligomers and curvilinear protofibrils concentrate largely on, and within, the upper‐leaflet of the bilayer. This causes a thickening in the appearance of the membrane, although Aβ insertion into the membrane is often described as causing thinning, because the density of lipids within the bilayer is reduced and replaced by Aβ. This causes the membrane to become more permeable. In contrast, cryoET images indicate fibrillar Aβ_42_ has little impact on the appearance of the liposomes, under these near native conditions, fibrils tend not to adhere or interact with the lipid bilayer.^[23]^ The phospholipid head groups do not attract the lateral surface of the Aβ_42_ fibril in an aqueous environment, at neutral pH.

**Figure 4:**
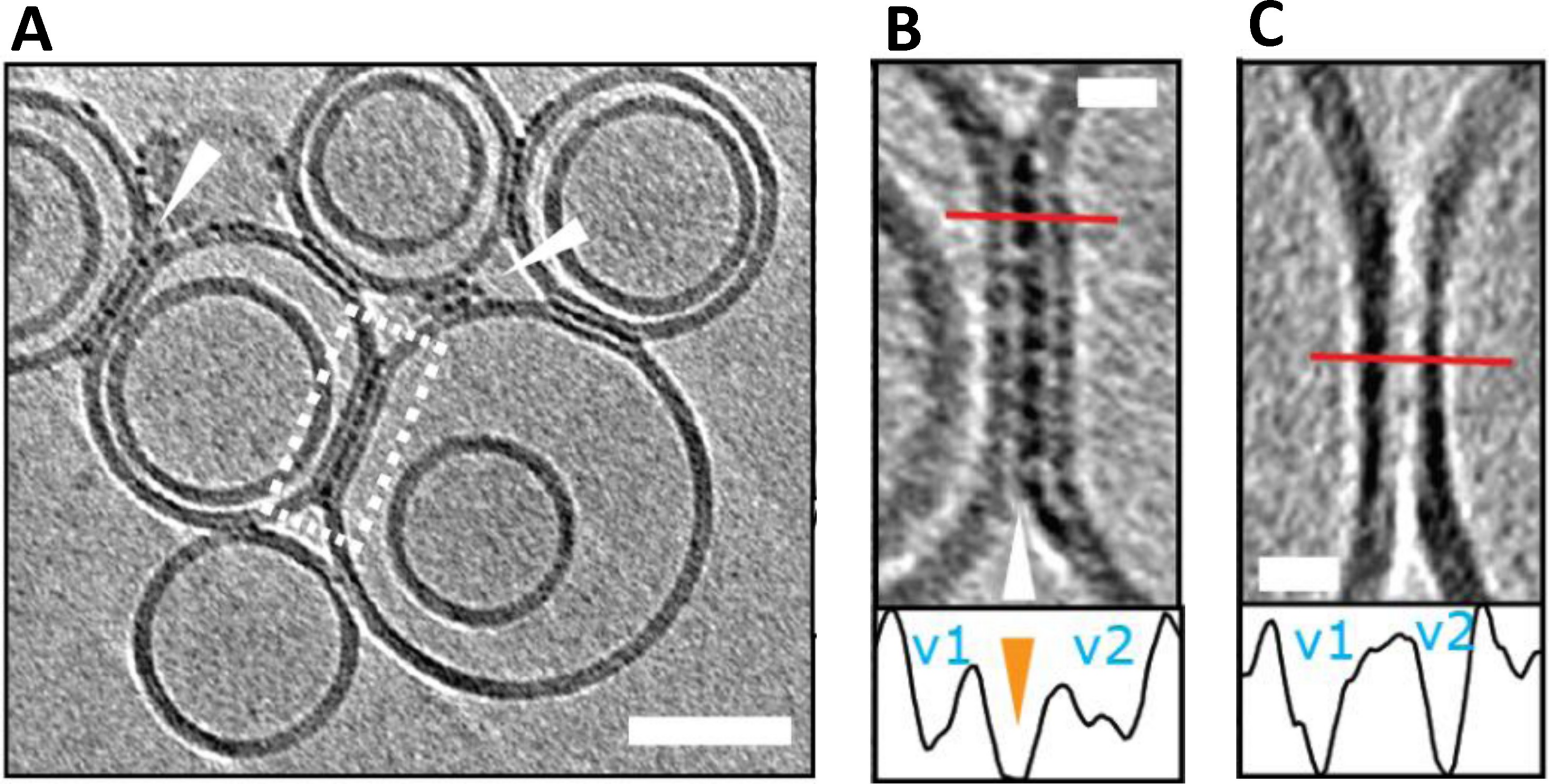
Liposomes can be linked together by Aβ42 protofibrils. (A) Aβ_42_ assemblies become localized to inter‐vesicular space and connect the membranes of neighbouring vesicles (arrowheads). In preparations of reduced levels of oligomer/protofibrils, Aβ_42_ decoration is not observed elsewhere on the vesicles. (B) Area rimmed in white is expanded. Profile plots along the red lines indicate the presence of additional density (orange arrowhead) between the two vesicles marked as v1 and v2. (C) Control experiment showing vesicles with no Aβ_42_ added. Note the absence of additional densities in areas where vesicles are in contact. The tomographic slices are 7.6 nm thick with scale bars: 50 nm, insets: 10 nm. Adapted from [23].

Interestingly, Aβ oligomers and curvilinear protofibrils become concentrated at interfaces between vesicles, this can result in a network of inter‐connected liposomes, Figure 4. The fluidity of the bilayer may facilitate the migration and clustering of Aβ_42_ oligomers at the interface, which are stabilized by binding across two membrane surfaces. We can speculate this same behavior will occur across the synaptic cleft. The gap across the synaptic cleft is 20–40 nm, similar in length to the Aβ curvilinear protofibrils. It seems reasonable to predict Aβ oligomers and curvilinear protofibrils once bound to the membrane will migrate and become concentrated at the synapse, causing it to become clogged with Aβ. This behavior might account for the reported loss of synaptic connections, which is a key early feature of AD pathology.^[8]^

CryoET imaging of synthetic vesicles suggests Aβ oligomers remain predominantly on the outer leaflet of the bilayer and do not traffic internally.^[23]^ In vivo, Aβ oligomers trafficking into the cytosol may occur via an additional membrane protein, indeed receptor mediated cellular internalization of Aβ by the cellular prion protein (PrP^C^) has been reported.^[67]^ Monomeric and dimeric Aβ are too small to be imaged by cryoET but have been reported to cross the bilayer.^[68]^

Membrane composition, in particular GM1‐ganglioside, has been shown to have a significant role promoting Aβ‐membrane interactions, GM1‐ganglioside is a lipid mostly found in the outer‐leaflet of neuronal plasma‐membranes.^[63b]^ The affinity of Aβ for GM1 is elevated relative to other lipids.[^65b^, ^69^] TEM and cryoET studies indicate Aβ_42_ oligomers have a reduced affinity for synthetic bilayers which lack physiological amounts of GM1‐ganglioside (*ca* 2 % by weight).^[23]^ It is suggested that the enhance affinity may be due to the hydrogen bonding capacity of the hydroxyls in the glycolipid headgroups.^[70]^ Cholesterol levels have been associated with the incidence of AD^[71]^ and cholesterol is suggested to influence membrane disruption by Aβ.^[72]^ However, cholesterol levels in synthetic bilayers do not appear to impact the Aβ oligomer disruption of membranes.[^23^, ^73^] Membrane associated cholesterol may act on Aβ in other ways, for example, accelerating oligomer and fibril formation.^[48]^

Cell culture in resin has been studied using scanning tomographic TEM, in this study it was possible to image individual fibrils in 3D, although protofibrils were not observed.^[74]^ As with isolated vesicles there is minimal interaction of the fibrils with the plasma membrane.^[74]^ Images of bundled Aβ fibrils resembling plaques hints at the future possibility of using cryoET to image plaques ex vivo.

CryoET has been used to study the interaction of other amyloid fibrils with lipid‐bilayers. Fibrils from the Huntington's disease protein have recently been imaged by cryoET.^[75]^ In addition, cryoET images have shown the ends of β_2_M fibrils distort the shape of liposomes.^[76]^ Heavy metal‐stained tomographic imaging of Serum Amyloid‐A fibrils have also been reported.^[77]^

### 4.2 AFM Imaging of Supported Lipid Bilayers

Atomic Force Microscopy (AFM) has been used to image the impact of Aβ assemblies on the lipid‐bilayer structure, supported on a surface such as mica (SiO_2_).[^25^, ^65^] Tapping‐mode AFM can provide nanometre‐scale membrane topography (sub‐nanometre in the z‐axis).^[78]^ The surface structure can be probed in air or liquid.^[65c]^ AFM studies have used two broad approaches: (i) Supported lipid‐bilayers can be generated which are then challenged by Aβ preparations.^[25]^ (ii) Alternatively, Aβ can be mixed with the lipid before it is allowed to lay‐down on the mica support.^[79]^ The latter has been employed to image Aβ annular oligomers surrounded by lipid bilayer.[^32^, ^59^] In addition, AFM has imaged the morphology of fibrils as they assemble in the presence of supported lipid bilayers.[^65a^, ^80^]

**Figure 5:**
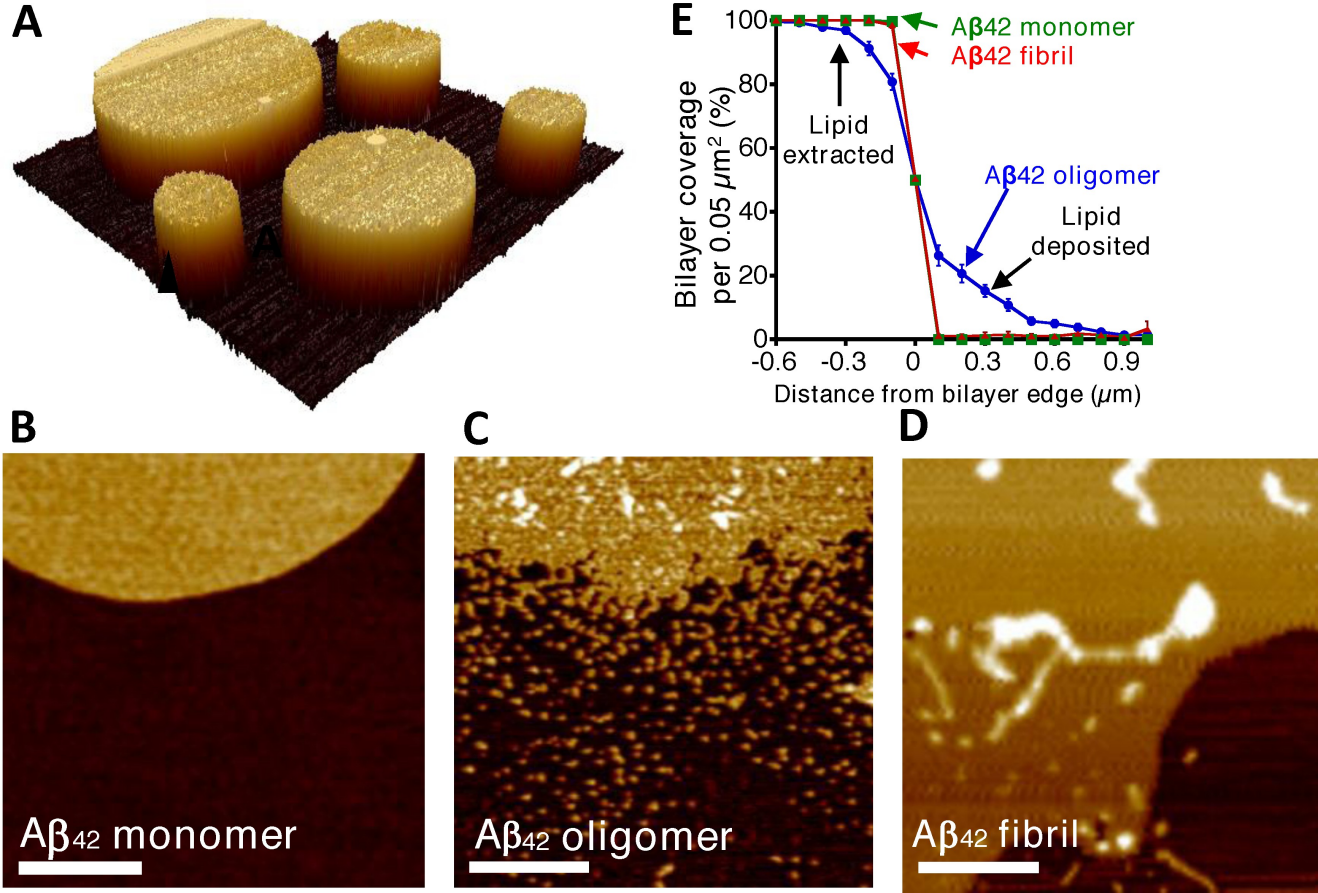
AFM imaging indicates Aβ_42_ oligomers have a detergent‐like effect on the supported lipid bilayer, which is not seen for Aβ_42_ monomer or fibrils. (A) AFM topographical image of lipid bilayer supported on mica surface. (B) AFM images are shown for mica‐supported lipid bilayers on exposure to Aβ_42_ monomer. (C) Aβ_42_ oligomers. (D) Aβ_42_ fibrils. Scale bar 500 nm, height scale range 12 nm. (E) Percentage of bilayer coverage at the edge of a lipid bilayer, exposed to Aβ_42_ monomer (*green*), oligomer (*blue*), and fibril (*red*). Each data point represents an average percentage of bilayer coverage within a 0.1×0.5‐μm region. There were 20 measurements per data point, measured across three separate mica‐supported lipid bilayer preparations. *Error bars*, S.E. Adapted from [25].

Lipid bilayers can readily be formed on the mica support, the lipid head‐groups align themselves on the surface of the hydrophilic mica, this is followed by the hydrophobic tail of the upper‐leaflet of the bilayer to create a remarkably flat and even surface. A lipid mixture lays down on the mica surface to form discs or ‘islands’ of lipid bilayer, 5 nm in height above the mica, Figure 5A. The impact on the lipid bilayer by both Aβ_42_ and Aβ_40_ isoforms have been studied in three distinct assembly states: monomers; prefibrillar heterogeneous oligomeric assemblies, and predominately mature fibrils. Largely, in agreement with the related cryoET imaging study^[23]^ AFM indicates these three preparations have very different impacts on the supported lipid‐bilayer,^[25]^ Figure 5B,C,D. The membrane islands, of PC:cholesterol:GM1 (68 : 30 : 2 % by weight) are unaffected by the presence of monomeric Aβ_42_. In contrast, the edges of the lipid island are perturbed by the presence of Aβ_42_ or Aβ_40_ oligomers. The effect has been likened to the action of a detergent where lipids are extracted from the membrane; a ‘halo’ of lipid is deposited close to the lipid island bilayer. Quantification of the extraction and deposition of the lipid can be used to compare the effect of different Aβ assembly forms from multiple preparations, Figure 5E.

The action of the Aβ oligomers, extracting lipids from the upper‐ and lower‐leaflet can produce holes in the supported lipid‐bilayer.[^25^, ^65b^] These holes are relatively large; typically, 50 nm in diameter,^[25]^ and if these were to occur in a cellular membrane, they would represent rupture of the cell. These holes should not be conflated with ion channel pores that can be formed by Aβ. Aβ ion channels, (described in the next section) are much smaller, typically 2 nm in internal diameter. It is notable that when vesicles (LUVs) are incubated with Aβ oligomers these large holes are not observed in related cryoET studies.^[23]^ Although lipid extraction by Aβ may occur within the vesicles the fluidity of the membrane will allow the space left by extracted lipid to be filled, but the lipid on the mica support may be less fluid leading to the holes observed.

The impact of mature fibrils on the supported lipid bilayer is less pronounced and the detergent‐like effect was not observed for Aβ fibrils.^[25]^ However, AFM images do indicate fibrils laterally associate and embed into the upper‐leaflet of the bilayer. This embedding of the fibril is indicated by measuring the height of the membrane and fibril, above the mica surface.^[25]^ This behavior is not apparent in vesicles imaged by cryoET in water. Thus, the lateral‐embedding along the length of the fibril, observed in the AFM studies, might occur because of water removal from the surface in these studies.

The impact of Aβ on supported lipid‐bilayers has also been studied in liquid. This has facilitated imaging membrane disruption over time, high temporal resolution indicates the membrane is disrupted by Aβ oligomers within seconds, while fibrillar Aβ_42_ has minimal impact on the bilayer.[^65c^, ^73^] Supported lipid‐bilayers containing POPC/SM/cholesterol/GM1 are particularly vulnerable to Aβ oligomers.[^65c^, ^73^]

The importance of the lipid‐bilayer composition in effecting Aβ interactions has been highlighted by a comparison of pure POPC with POPC/cholesterol/GM1 supported lipid‐bilayers (slb).^[81]^ With cholesterol added, the lipid‐bilayer can phase‐separate to form distinct microdomains or rafts. It is suggested this phase separation may create deficits which enable Aβ oligomers to insert and disrupt the membrane, while pure POPC bilayers remain unperturbed by Aβ.^[81]^

Living hippocampal neurons have also been studied by AFM, membrane elasticity measurements using force‐indentation curves indicate a reduction in membrane stiffness in the presence of Aβ oligomers. Interestingly, only the aged neurons were softened by the presence of both Aβ_40_ and Aβ_42_ oligomers, and it was shown that these aged neurons have depleted cholesterol levels.^[56c]^ Tapping‐mode AFM images were not able to show marked differences in the appearance of the neurons but were restricted by resolution and incubation times.^[56c]^ A lipid mixture from brain total lipid extract (BTLE) showed a decrease in the Youngs moduli (or stiffness) of the bilayer, in the presence of Aβ_42_ oligomers.^[79]^ Similar reductions in stiffness of the supported lipid‐bilayer have been reported to be induced by the presence of Aβ oligomers but not fibrils.^[82]^ Using force indentation measurements it has been shown a range of amyloid forming proteins reduce membrane stiffness, and therefore a shared mechanism of membrane softening has been proposed.^[83]^

### 4.3 Negatively Stained TEM of Liposomes

TEM has also been used to image Aβ‐vesicle interactions.[^23^, ^25^, ^46a^] Although heavy‐metal staining can cause artefacts such as flattening of spherical objects due to sample drying, the conclusions drawn from liposome images obtained in the presence of Aβ are in broad agreement with the more native conditions used in cryoET. Aβ monomers have no detectable impact on the integrity of membrane vesicles. While addition of preformed Aβ oligomers cause widespread curvatures of the lipid vesicle bilayer.[^23^, ^25^] The disruption of the membrane by Aβ oligomers is even more apparent than with native cryo conditions, heavy metal staining, especially uranyl‐acetate, indicates budding and possible extraction of lipids from the bilayer in the presence of Aβ oligomers.^[23]^ There are also ruptures of the bilayer observed in the presence of Aβ_42_ oligomers kinetically trapped by the addition of Cu^2+^.^[46a]^

Negatively stained TEM images show some interactions of Aβ fibrils with the lipid bilayer causing some distortion in the curvature of the bilayer, but this is less widespread than for the oligomers.^[25]^ The locus of the effects is typically centered at fibril ends rather than on the lateral surface, similar effects have been reported for amyloid fibrils of β_2_M when imaged by cryoET.^[76]^

CryoEM and TEM have also been used to image vesicles generated after incubation with Aβ monomers during fibril formation, rather than using preformed oligomers or fibrils. Interestingly, Aβ oligomers and fibrils formed in the presence of an inhibitor of secondary nucleation do not disrupt the membrane, even though fibrils are formed from monomer. In contrast, if oligomers and fibrils are permitted to form via secondary nucleation, significant disruption of the membrane is observed.^[84]^ Complementary cell viability studies suggests that the pathway to fibrils impacts cytotoxicity.

### 4.4 NMR and MD Simulations in Lipid Bilayers

Discussions so far have described Aβ‐membrane interactions imaged at the nanoscale. The cryoET and AFM techniques are sufficient to resolve the inner‐ and outer‐leaflet of the membrane but are not able to resolve individual amino acids. NMR and molecular simulations are currently best placed to fill this gap and achieve atomistic resolution. A careful solution NMR study has shown monomeric Aβ_40_ binds weakly to the membrane surface forming loose transient helical structures,^[85]^ this is in general agreement with MD simulations of Aβ monomer interactions.^[86]^

**Figure 6:**
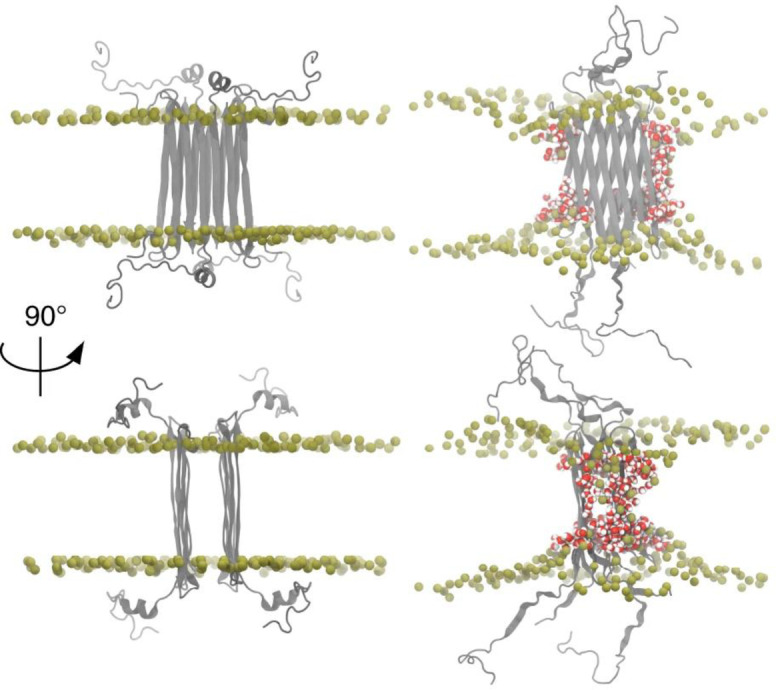
Aβ_42_ octamers structure in lipid environment. Octameric Aβ_42_ structure based on solution NMR within a DPC micelle, followed by MD simulations in phospholipids. Aβ_42_ is shown in grey, DPPC headgroup phosphorous atoms are shown in tan. Water in red/white highlights Aβ induced membrane permeability. Adapted from [58].

Solution NMR studies with small membrane micelles have shown Aβ octamers can form an anti‐parallel β‐sandwich structure within a membrane, Figure 6.[^57^, ^58^] An Aβ‐tetramer structure has been determined by solution NMR, embedded with a dodecyl‐phosphocholine (DPC) micelle. Using TROSY based assignments a β‐sheet 3D structure has been identified from chemical shift indexing (CSI) and NOE constraints. At very high Aβ concentrations (450 μM) it was possible to study an octameric structure consisting of a β‐sandwich of two tetramers. MD simulations and electrophysiology measurements indicate this structure is permeable to ions, Figure 6. The relationship of this octamer to larger annular oligomers, perhaps containing 16–24 Aβ molecules (as suggested by MD and AFM studies) is not clear (see section 5.2).

MD simulations have been used to probe both monomers, dimers, protofibrils and fibril interactions with various simulated lipid‐bilayers, see reviews.^[87]^ Aβ transmembrane structures have been modeled, and the simulations indicate permeability to water, Ca^2+^ and other ions. MD indicates Aβ favours the formation of β‐sheet containing tetrameric and hexameric structures in the bilayer.^[88]^ Similarly, ion mobility mass spectrometry supports the formation of hexametric structures in a membrane mimicking environment, and are consistent with β‐barrel formation.^[89]^ Simulations by Jang et al. generate Aβ β‐barrel structure imbedded in the bilayer, with many of the properties imaged by AFM of annular oligomers and Aβ ion channel‐pore conductance's,^[90]^ described in the next section. More recently molecular dynamics simulations have been used to model U shaped Aβ trimers as the minimal oligomer size to insert into the bilayer.^[91]^ All atom MD simulations indicate that Aβ_42_ trimers can form small β‐barrels within the bilayer, which are capable of forming internal pores large enough to be accessed by water and Ca^2+^ ions.^[92]^

## 5 Aβ Ion Channel Pores

Membrane permeability can also occur by formation of ion‐channel pore assemblies of Aβ that span the lipid bilayer. The ion‐channel hypothesis as a mechanism for Aβ toxicity was first proposed by Arispe et al. in 1993.^[21]^ It remains an important element in our understanding of AD pathology.[^21^, ^22^, ^59^, ^93^]

### 5.1 Conductance Measurements

**Figure 7:**
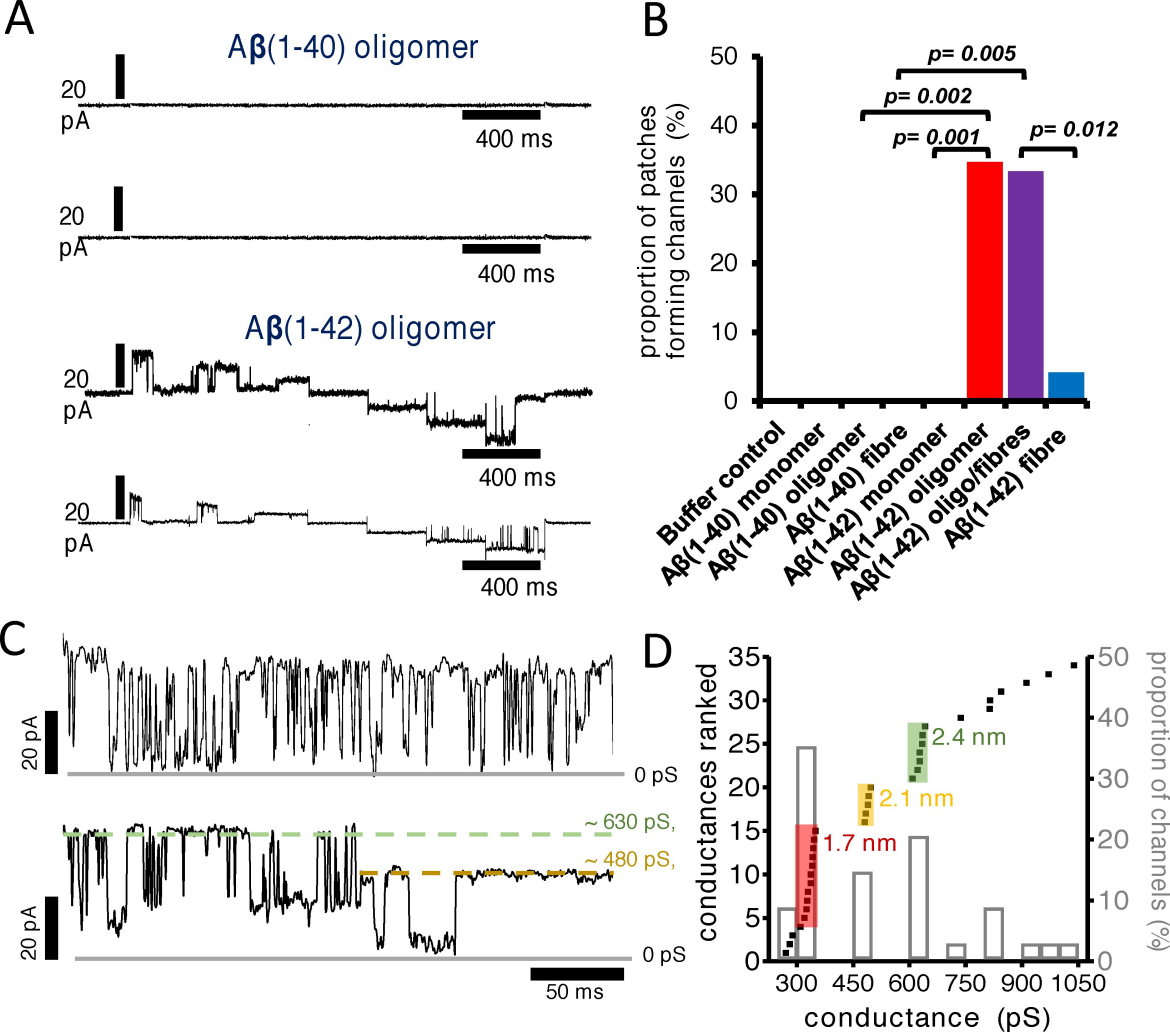
Aβ ion channel pore conductance. (A) Ion channel conductance recordings for Aβ oligomers. Current was recorded at each potential (stepped protocol between −60 mV and +60 mV), and two representative traces recorded for Aβ(1–40) and Aβ(1–42) oligomers. (B) The proportion of membrane patches that formed channels for monomeric, oligomeric, and fibrillar Aβ(1–40) and Aβ(1–42). Channel formation was significant for Aβ(1–42) oligomer and oligomer‐containing fibril samples. (C) Aβ(1–42) channels exhibit two distinct conductance behaviors: Rapid flicker between low and high conductance states (*top*) and step transitions between conductance levels (*bottom*). Measured conductance states are highlighted by dashed lines. Gray solid lines represent zero baseline current. Currents are recorded from membrane patches held at a +60 mV. (D) Conductance distribution in the presence of Aβ(1–42) oligomers. The primary *y axis* represents a rank order of conductance with increasing magnitude for 34 channels. Three discrete channel subtypes are highlighted with estimated pore diameter. A secondary *y axis* indicates proportion of channels formed (bar‐chart) within a 50‐pS bin size. The most common conductance observed is 300–350 pS; 35 % of channels. Adapted from [22].

The initial membrane conductances were observed by reconstituting synthetic lipid‐bilayers around Aβ assemblies and recording voltage‐patch‐clamp conductance.^[21]^ Using this approach, no notable difference in the channel‐forming properties between Aβ_40_[^32^, ^94^] and Aβ_42_ were reported.[^57^, ^59^, ^95^] Conductance measurements indicate a particular set of features: (i) Single Aβ channels are large, with conductance usually greater than 250 pS. (ii) They remain open for long periods of time, typically more than 500 ms. (iii) The spontaneous voltage‐independent activation can transition between multiple conductance states.[^93^, ^96^] The large Aβ channels are unregulated, flexible and have uncertain selectivity which suggests they should be viewed as flexible pore like entities. More recently it has been reported that when cellular membrane patches were used, and Aβ assemblies were permitted to diffuse and insert into the extra‐cellular surface of the membrane, only oligomeric preparations of Aβ_42_ cause single ion channel currents, Figure 7A,B, while monomeric and isolated fibrils of Aβ_42_ did not form channels.^[22]^ All assembly forms of Aβ_40_, including oligomers, were unable to insert into the cellular membrane and form ion channels, Figure 7B. This suggests that only prefibrillar assemblies of Aβ_42_ have properties which allow cell membrane insertion and channel formation. This strengthens the ion‐channel hypothesis, by making a direct link between the specific ability of Aβ_42_ oligomers to form ion channels and observed AD pathology, which points to Aβ_42_ not Aβ_40_ toxicity.

The magnitude of the conductance can be used to estimate the size of openings across the membrane. Conductances have been reported to group into three main channel subtypes: ca. 340 pS; 490 pS and 630 pS, Figure 7C,D. This suggests an approximate internal pore diameter of 1.7 nm, 2.1 nm and 2.4 nm; assuming a channel length within the lipid bilayer of 7 nm.^[22]^ These implied pore diameters closely match the sizes indicated for annular oligomers as imaged by AFM and negatively stained TEM images, as well as determined from molecular dynamics simulations, described in the next section.[^32^, ^59^, ^90^, ^97^] This makes an important link between observed oligomer structures and the size of the conductance. Serra‐Batiste et al. reported 200 pS conductance for Aβ_42_ pores, and using a similar approach suggested an internal pore diameter of 0.7 nm.^[57]^ This internal diameter was based on a channel length of 3 nm, not 7 nm assumed by Bode et al. for the annular structures. Transitions between conductance states, Figure 7C, indicate Aβ ion channels are dynamic and can change in pore dimensions, which is supported by multiple sizes of Aβ annular oligomers structures reported.[^32^, ^59^, ^97b^, ^98^] MD Simulations also indicate the dynamic nature of the barrel shape structures.[^90^, ^98^] In addition, different conductance behaviors are observed for Aβ channel‐pores, exhibiting a flicker or a continuous appearance, Figure 7C. These differences may also reflect changes in Aβ oligomer structure and flexibility.

Early studies suggested that the Aβ channel pores were ion selective,[^21^, ^32^, ^93^, ^94^] however other studies do not support this observation,^[22]^ the large size and flexibility of the pores might suggest robust ion selectivity is unlikely. Zn^2+^ ions can block the channel conductance, the mechanism is not clear but may be associated with disruption of Aβ assembly^[46c]^ and so may restrict Aβ from forming annular oligomers.

Various truncated forms of Aβ have also been studied, N‐terminally truncated Aβ with pyroglutamate Aβ_(pE3–42)_ exhibits heightened conductance.^[99]^ Aβ_(9–42)_ and the p3 peptide, Aβ_(17–42)_, have also been reported to produce ion channels.[^97a^, ^100^] In contrast others have described ion channel conductance for Aβ_(25–35)_ but not Aβ_(17–42)_ or Aβ_(1–28)_.^[101]^

The lipid membrane composition in synthetic bilayers also impacts the channel conductance. Cholesterol at 15 % w/w of lipid, promotes Aβ insertion and channel formation, while at higher or lower levels of cholesterol a reduction in channel conductance is reported.^[102]^ Conductance across bilayers generated from BTLE are compared to bilayers formed from a DOPS/POPE lipid mixture. A larger conductance across the bilayer was observed when more DOPS was present in the membrane, while it was noted that changes in the AD brain have increase quantities of DOPS phospholipid.^[100]^

If channel formation is indeed key to cytotoxicity of Aβ_42_ then molecules that block, stop their formation, or insertion, should be a powerful therapeutic approach.[^93^, ^96^] Promisingly, a diphenylpyrazole molecule blocks Aβ channels and rescues disease phenotypes in a mouse model for amyloid pathology.^[103]^

Other oligomers associated with amyloid diseases can also form ion‐channels, for example, α‐synuclein from Parkinson's disease^[104]^ and oligomers of β_2_‐microglobulin in dialysis related amyloidosis.^[105]^

### 5.2 Channel Resembling Annular Oligomers Aβ Structures (Little Rings)

**Figure 8:**
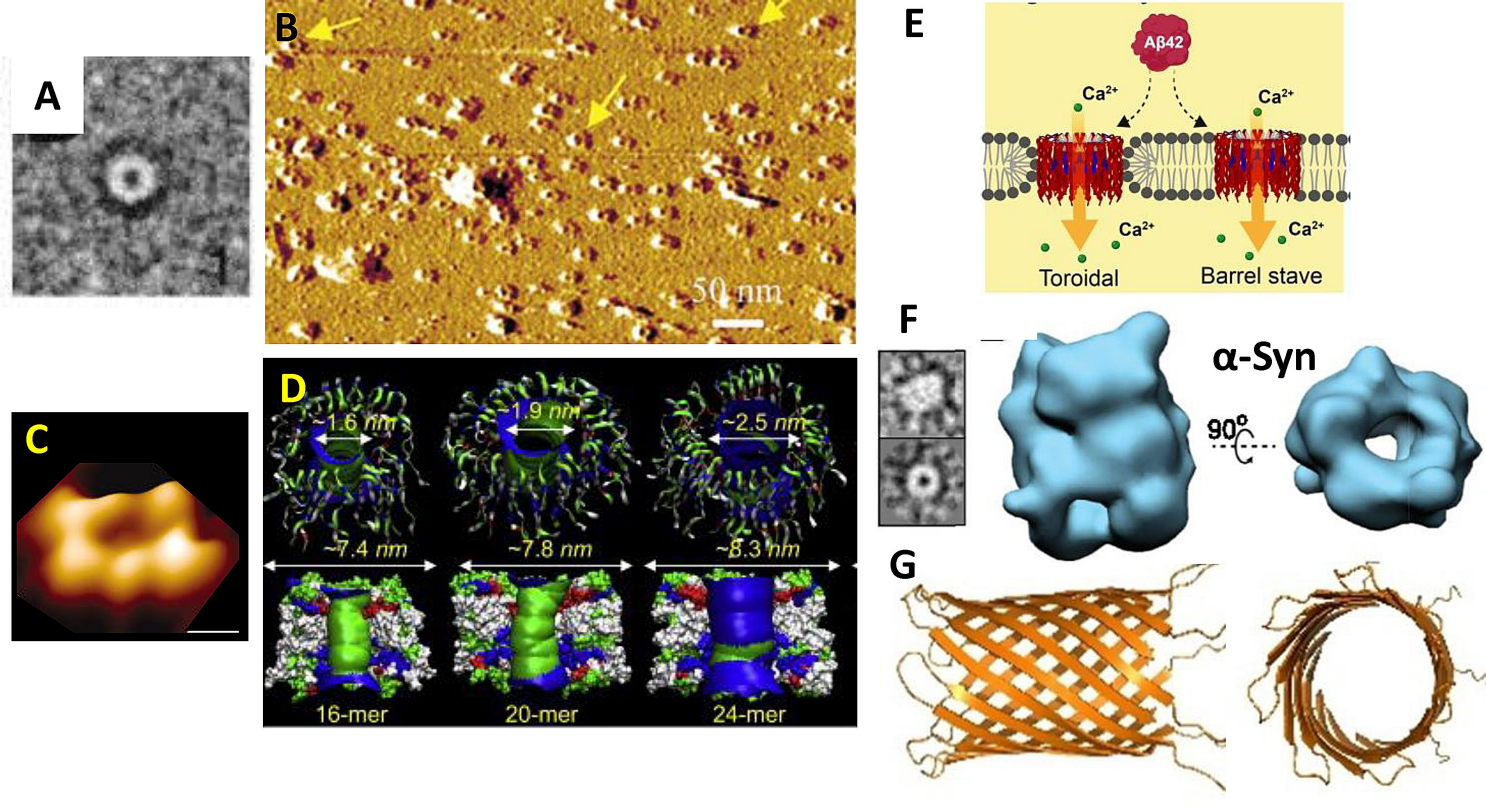
Annular Oligomers (A) Negatively stained TEM 2D class averages of Aβ42 (arctic mutant).^[98]^ (B) Many annular oligomers of Aβ_42_ embedded within the supported lipid bilayer, imaged by AFM[^32^, ^59^] (C) A single annular oligomers of Aβ_42_ imaged by AFM.^[32]^ (D) MD simulation showing possible β‐barrel conformation, a series of structures (1.6–2.5 internal diameter from 16 to 24 Aβ molecules.^[90]^ (E) Possible modes of insertion of annular oligomers within the lipid bilayer; barrel stave or toroidal.^[25]^ (F) CryoEM 3D structures of alpha‐synuclein shows a similar annular structure.^[106]^ (G) CryoEM structure of Aβ heptameric β‐barrel within a large protein scaffold.^[107]^

Channel–pore resembling structures were first reported two decades ago by negatively stained TEM images, Figure 8A, for both Aβ but also assemblies for αSyn from Parkinson's Disease.[^97b^, ^98^] Using atomic force microscopy (AFM), Lal et al. have imaged similar pore‐resembling structures composed of Aβ inserted in supported lipid‐bilayer, with the channel consistently aligned upright, so as to suggest the pore can span the membrane Figure 8B,C.[^32^, ^59^, ^97a^, ^108^, ^109^] These images are generated by first mixing lipid with Aβ and then allowing these to form a lipid bilayer on the mica surface, with Aβ oligomers incorporated within the lipid bilayer. These annular oligomers (meaning ‘little rings’) have a typical external diameter of ca 10 nm and form annular donut‐ring structures with an internal diameter of 1–2 nm,[^32^, ^59^] shown in Figure 8. These structures protrude above the membrane by 1 nm. Negatively stained TEM 2D class averages and AFM images suggest more than one size of annular oligomer can form, with internal diameters from 1–2 nm and 6–9 nm external diameter,[^97b^, ^98^] this agrees with the variable conductance measured.

Molecular simulations predict dynamic β‐barrel structures^[110]^ containing 16 to 24 Aβ monomers with an estimated internal pore diameter of between 1.6 and 2.5 nm respectively, Figure 8D,^[90]^ and also for N‐terminal truncated Aβ.^[111]^ These diameters closely match the diameters calculated based on conductance measurements for Aβ_42_ oligomers.^[22]^ Larger pore‐like structures with up to 6 nm pore opening and 25 nm outer diameter[^97b^, ^112^] might produce larger conductances which are also observed. Significantly, ex vivo annular oligomer assemblies with a 2.5‐ 4 nm pore diameter have been imaged from AD model mice and human AD frontal cortex.^[113]^

Two modes of membrane insertion for annular oligomers could occur. A toroidal structure, where the Aβ barrel interacts with both the charge headgroups at the surface and the hydrophobic acyl chains within the middle of the membrane. Alternatively, the stave type structure might be formed, where the membrane edge is capped by headgroups, so the β‐barrel only has contact with the charged part of the membrane, Figure 8E. The observation that the annular oligomers consistently align upright in the supported lipid bilayer, Figure 8B, might hint at a toroidal insertion. It is suggested that free phospholipids, with short chain‐lengths and μM critical micelle concentrations (CMC), can bind to the annular oligomers and promote membrane insertion.^[114]^

Cryo‐EM 3D structures of Aβ oligomers are a challenge for several reasons, oligomers are highly heterogeneous, they are flexible, and they are small (30–100 Å). One approach has been to fuse a larger protein with Aβ. Using an α‐hemolysin scaffold, Aβ retains its channel‐pore electrophysiology properties; displaying conductances between 200–500 pS. The particles generated are homogeneous with a clear heptameric structure and C7 symmetry. The Aβ sequence within the scaffold forms a β‐barrel structure with seven Aβ molecules (PDB 7O1Q), with a length of 35 Å and an internal diameter of 27 Å (Figure 8G)^[107]^ This β‐barrel structure appears to be smaller than the annular structures, also shown in Figure 8. Annular structures have been generated by single particle analysis and 3D reconstruction for αSyn from Parkinson's disease,^[106]^ Figure 8F. These 3D structures, obtained from a subset of just 7 776 single particles, are sufficiently resolved to describe the internal pore of the annular oligomers.^[106]^ Like Aβ, α‐synuclein can produce ion channel conductance across membranes.[^97b^, ^104^, ^115^, ^116^] A shared mechanism of ion pore cytotoxicity has therefore been suggested for annular oligomers of amyloid proteins.[^32^, ^97b^] Indeed, annular oligomers have been image by AFM inserted in the lipid‐bilayer for as many as six different amyloid proteins.^[32]^

## 6 Membrane Permeability and Cytotoxicity

### 6.1 Permeability in Synthetic Models and Cells

The permeability of the membrane in the presence of Aβ assemblies have been measured by various techniques. Membrane conductance, due to a flow of Ca^2+^ and other ions across lipid monolayers has been described.[^24^, ^117^] The conductances reported across large areas of lipid‐monolayer are small, caused by a general leakage of ions, and are not from the larger annular oligomers, that cause distinct ion‐channels.^[118]^ This increase in permeability is observed for oligomers of both Aβ_42_ and Aβ_40_.[^24^, ^117^] It seems reasonable to assign these permeability effects to the Aβ insertion and carpeting by small oligomers (trimers‐hexamers) and curvilinear protofibrils observed for both Aβ_40_ and Aβ_42_.^[23]^

**Figure 9:**
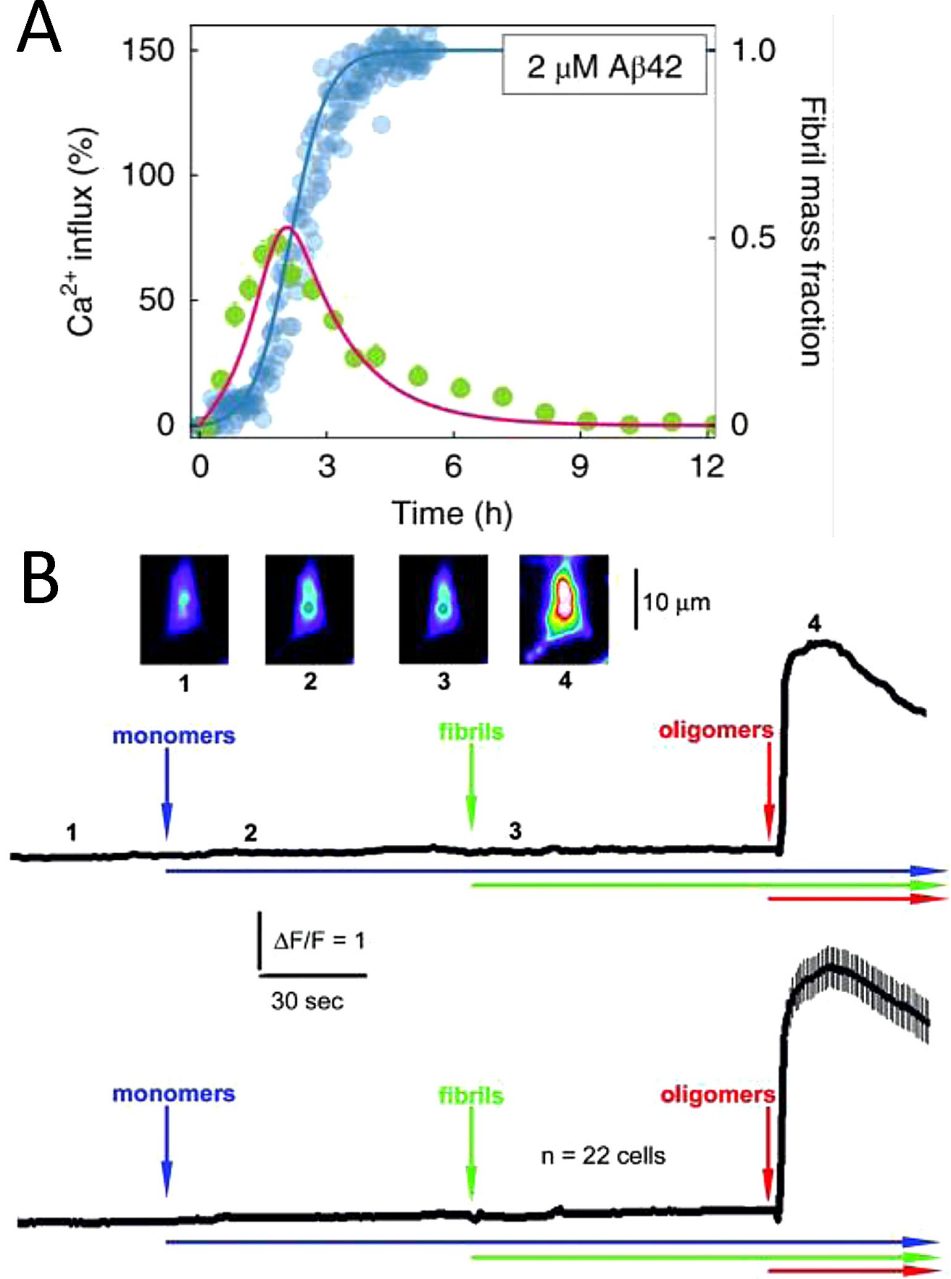
Membrane permeability to Ca^2+^ ions in vesicles and cellular systems. (A) Fibril formation monitored alongside Ca^2+^ influx into lipid vesical. Direct relationship between prefibrillar Aβ_42_ oligomer levels and Ca^2+^ influx.^[119a]^ (B) Ca^2+^ influx into cells detected by Fluro‐2 only observed for Aβ oligomers added to the extracellular culture. The average of 22 individual cell recordings is shown in the lower trace.^[121]^

Others have developed fluorescence microscopy methods to monitor Ca^2+^ influx into single synthetic vesicles.^[119]^ These studies have indicated a direct correlation between membrane permeability and the kinetic assembly of Aβ_42_ oligomers via secondary nucleation, Figure 9A.^[119a]^ The Ca^2+^ influx in synthetic vesicles indicates that the permeability observed is independent of endogenous membrane proteins such as glutamate receptors or cellular calcium ion channels (see section 8). This does not rule out Aβ receptors as a mechanism for impaired calcium homeostasis but could be in addition to direct membrane permeability. Bulk membrane permeability of vesicles has also been measured using the release of large fluorescent dyes, calcine,^[120]^ or carboxy‐fluorescein^[114]^ encapsulated within synthetic vesicles (LUVs). While influx of the smaller Ca^2+^ ions into vesicles has been monitored by encapsulated Fluo‐2 dye.^[114]^

Ca^2+^ influx in cellular systems have been detected using cells loaded with Ca^2+^ sensitive fluorescent dye (FLIPR‐Molecular Devices).^[13d]^ Similarly, elevated Ca^2+^ levels for individual cells have been detected induced by Aβ_42_ oligomers, but not fibrils or monomers, using a Ca^2+^ sensitive florescent dye (Fluo‐3)^[121]^ and (Fluo‐2), Figure 9B.^[59]^ Typically, the influx of Ca^2+^ occurs within less than a minute after the addition of Aβ_42_ oligomers. The cellular fluorescent signal then subsides within a few minutes.[^59^, ^121^] While ion‐channel conductance takes longer for annular‐oligomer insertion into the membrane; typically 5–10 mins.^[22]^ In a study in which the neuronal plasma membrane is either depleted or elevated with levels of GM1, a direct relationship between Aβ_42_ oligomers binding to the membrane and Ca^2+^ influx into neurons has been demonstrated.^[69a]^ Prefibrillar soluble aggregates formed at different stages of assembly exert cytotoxicity through different mechanisms. Small oligomers, ca 3 nm high, cause Ca^2+^ influx into vesicles, while larger assemblies cause an inflammatory response.^[122]^

### 6.2 Cellular Toxicity

An increase in membrane permeability leads to a loss of cellular homeostasis and an influx of Ca^2+^, which can culminate in cell death.[^18b^, ^18c^, ^18d^] Cell viability essays have long been used as a measure of Aβ cytotoxicity.[^13c^, ^15a^] Early studies have reported some cytotoxicity for fibrils and monomers, but this may simply reflect the dynamic heterogeneous nature of Aβ preparations and the presence and formation of oligomers within the samples studied. Careful studies have shown Aβ oligomers and not fibrils are the most cytotoxic.^[13c]^ Using 5 μM Aβ_42_ and SH‐SY5Y cells, both cell viability and cell toxicity assays have shown that larger curvilinear or annular protofibrils are a little more toxic to cells than smaller oligomers.^[13d]^ This comprehensive study also showed that disrupted cellular homeostasis was apparent in radical oxygen species (ROS) production and lipid membrane peroxidation. Oligomers and particularly Aβ_42_ curvilinear protofibrils perturbed these markers of cellular homeostasis.^[13d]^ Interestingly, both Aβ_40_ and Aβ_42_ are reported to be cytotoxic, although Aβ_40_ is less toxic than Aβ_42_.[^13c^, ^15a^] This falls in‐line with the observation that both Aβ_42_ and Aβ_40_ are capable of disrupting and carpeting lipid bilayers.^[23]^ The difference in cytotoxicity between Aβ_42_ and Aβ_40_ may reflect structural differences of these two isoforms or may simply be due to differences in the quantity of oligomers generated on the pathway to fibrils. Attempts to map regions of the Aβ_40_ sequence that form a toxic surface suggest a hydrophobic face, which includes residues 17–28 are key to Aβ’s cytotoxicity.^[123]^

## 7 The Impact of Lipid Membranes on Aβ Assembly

In addition to the impact of Aβ on membrane structure and permeability (Sections 4–6) there is much interest in how the presence of lipid‐bilayers can affect the rate of formation of oligomers and fibrils.^[17d]^ Indeed accumulation of amyloid deposits of Aβ peptide in the AD brain can be related to abnormal lipid metabolism.^[124]^ Using the fibril specific dye‐ ThT, fluorescence measurements have been used to investigate the rate of fibril formation in the presence of various lipid vesicles.[^48^, ^125^] Lipid bilayers have been reported to both accelerate and inhibit the rate of fibril formation. These seemingly conflicting observations appear to be dependent on the composition of the lipid bilayer.^[125b]^ In particular, the presence of cholesterol can accelerate primary nucleation, with the rate of fibril formation increasing 20‐fold.^[48]^

The impact of the phospholipid head‐group can certainly effect the rate of fibril formation, with zwitterionic lipid vesicles (POPC and POPE) causing fibrils to form faster than when in the presence of anionic lipids (POPS and POPG).^[125b]^ Others have also reported accelerated fibril formation in the presence of PC containing lipid vesicles: POPC; DOPC and DMPC.[^48^, ^80d^] However, an accelerating impact on fibril formation for PC containing vesicles is not universally reported.[^126^, ^127^] Furthermore, anionic DOPS has been reported to accelerate fibril formation to a greater extent than neutral DOPC.^[125a]^ Thus, a consensus as to the effect of charge and phospholipid head‐group is not yet resolved.

Globally fitting the fibril kinetic curves at different Aβ_42_ concentrations indicates that the acceleration of fibril formation in the presence of POPC is driven by changes in the rate of secondary fibril catalysed nucleation and fragmentation and is independent of primary nucleation.^[125a]^ This indicates the lipid bilayer can impact the surface of fibrils and their fragmentation. Analogous to this, a careful study of IAPP fibril‐formation indicates lipid‐bilayers can also accelerate the rate of IAPP secondary nucleation.^[128]^

Increasing the concentration of lipids relative to Aβ tends to reduce the fibril dependant ThT signal, this adds to the complexity of understanding the kinetics.^[129]^ The signal loss may be due to a reduction in the amount of Aβ monomer available to from fibrils or may be caused by the disruption of ThT detection. A study of preformed fibrils suggests ThT detection is not disrupted in the presence of lipid bilayer for phospolipides with long chains.^[125a]^ The length and saturation of the acyl‐chain may also influence fibril formation, in particular the short neutral phospholipid, DLPC, can reduce the ThT detected fibril signal, suggesting a reduction in the amount of Aβ monomer available to from fibrils, compared to the longer DOPC.^[80d]^ It is suggested fibril formation is influenced by the presence of free phospholipids, which are present at low concentrations in equilibrium with the lipid bilayer. The level of free phospholipid is dependent on each phospholipid's critical micelle concentration (CMC), this is affected by the lipid headgroup and alkyl‐chain. Comparison of a series of phospholipids (14 : 1; 18 : 1; 20 : 1 acyl‐chain lengths) show a clear trend in maximum ThT signal. PC(20 : 1) with a low nM CMC has little impact on ThT signal, while PC(14 : 1) with a μM CMC, will inhibit fibrils from forming.^[114]^

The influence of curvature of the membrane on fibril formation has also been explored by comparing the relative effects of SUVs with LUVs.[^48^, ^129^, ^130^] In addition, the combination and ratio of phospholipids and cholesterol, together with the formation of micro‐domains (rafts) can further complicate the influence of the membrane on Aβ assembly.

In addition to the effect of the lipid membrane on the kinetics of fibril formation, membranes can also perturb the fibril morphology,^[131]^ as indicated by AFM studies[^65a^, ^80^] and also a ssNMR structure of fibrils generated in the presence of lipids.^[132]^ With the immense complexity of lipid membranes, a clear picture of the impact they have on Aβ assembly and morphology is still emerging.

## 8 Aβ Membrane Protein Binding Partners

So far, this review has discussed the interaction of Aβ with the lipid‐bilayer for both synthetic and cellular systems, these interactions can be independent of membrane protein binding partners. There are also numerus membrane associated proteins identified as binding partners with Aβ, this in turn may affect cellular homeostasis and Aβ assembly. These can only briefly be described here, this controversial area has been reviewed elsewhere.[^17c^, ^29^, ^133^]

Laurén and colleagues focused attention on the cellular prion protein (PrP^C^). In a 200 000‐strong screen of a human cDNA library, PrP^C^ was shown to be the only high‐affinity binder of Aβ oligomers.^[28]^ Oligomeric forms of Aβ are bound to PrP^C^ in human brains.^[134]^ The cellular prion protein is a cell surface glycoprotein localized at synaptic terminals.^[135]^ PrP^C^ can heighten Aβ impairment of synaptic plasticity.^[136]^ In some mice models PrP^C^‐ Aβ interactions seem to be essential for neurotoxicity.[^28^, ^137^] Similarly, Aβ expressing *Drosophila* crossed with prion protein expressing flies have a pronounced AD phenotype, with a reduced longevity and disrupted circadian rhythms, not observed for the uncrossed Aβ or PrP^C^ expressing *Drosophila*.^**[47b]**^ The unstructured N‐terminal half of PrP^C^ binds to Aβ oligomers with nanomolar affinity.[^136^, ^138^] The mechanism of this neurotoxicity seems to be associated with the ability of the N‐terminal half of PrP^C^ to bind and trapped Aβ in an oligomeric form at the membrane surface.[^138c^, ^139^] This may be sufficient to mediate Aβ oligomeric toxicity, alternatively PrP^C^ may interact with other proteins such as the N‐methyl‐D‐aspartate receptor (NMDAR),^[140]^ Fyn kinase^[141]^ or mGlu5^[142]^ to heighten Aβ toxicity. Taken together these studies suggest PrP^C^ can mediate the toxicity of Aβ. For more details see the review by Collinge, et al.^[133b]^

Glutamate receptors have also been linked with Aβ induced toxicity and disrupted cellular Ca^2+^ homeostasis. NMDAR is linked with perturbed synaptic Ca^2+^ handling which causes synaptic depression and spine elimination.^[143]^ In addition, Metabotropic glutamate receptors (mGluR) are co‐localized with Aβ.^[144]^ Aβ oligomers cause Ca^2+^ release from the ER while NMDA receptors trigger excessive influx of Ca^2+^ into neurons which leads to Ca^2+^ toxicity. However, the mechanism underlying NMDAR dysfunction and AD is yet to be resolved.^[143]^ Aβ interaction with endogenous Ca^2+^ channels has also been suggested as a cause of elevated cellular Ca^2+^.^[18d]^ Although importantly, Aβ induced cellular Ca^2+^ influx is independent of plasma membrane Ca^2+^ channels.[^13d^, ^121^]

## 9 Summing Up and the Prospects for Therapeutics

**Figure 10:**
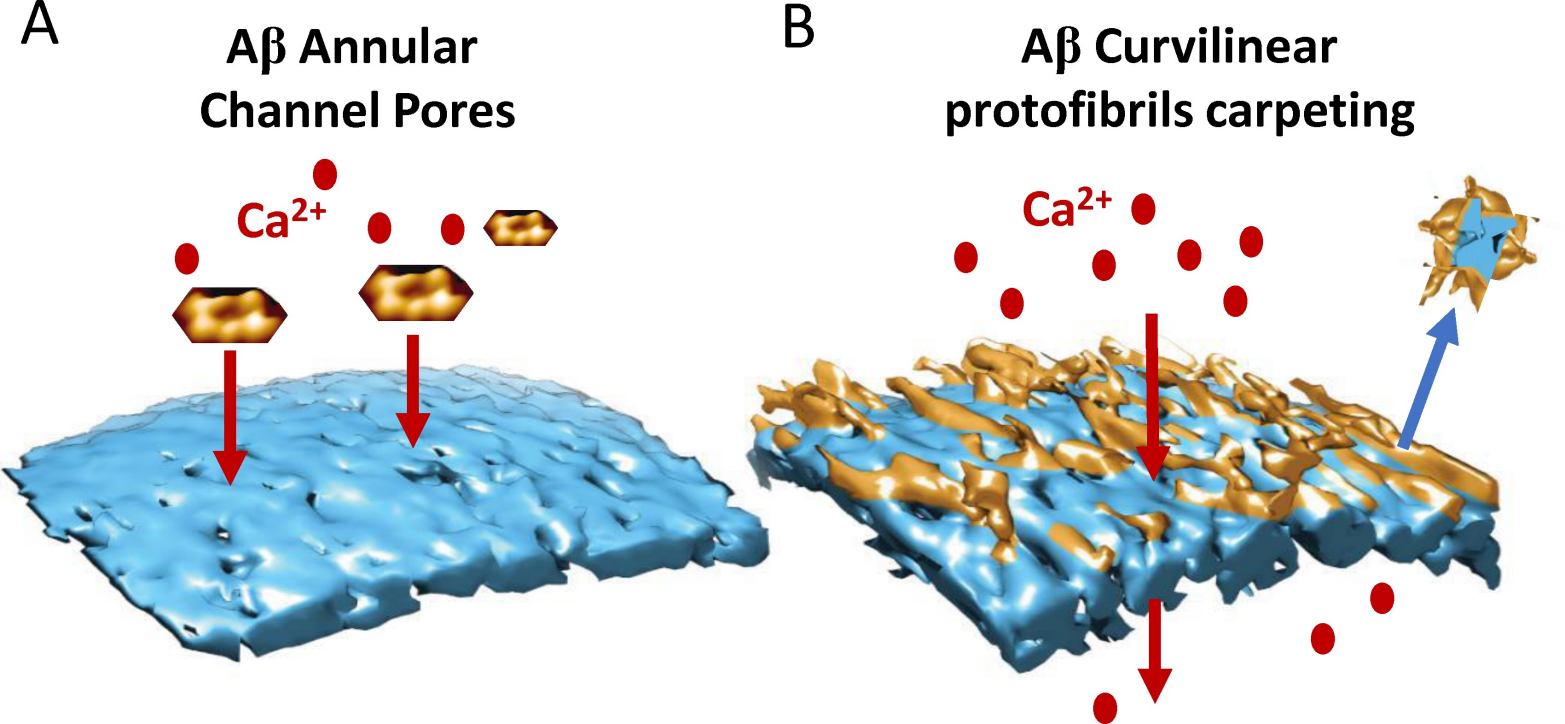
Summing up Aβ membrane interactions. (A) Aβ_42_ annular oligomers insert into the membrane and form ion Channel Pores; cartoon showing AFM images. (B) Aβ Curvilinear protofibrils and oligomers carpeting and inserting into the bilayer, causing membrane permeability and lipid extraction, describe as a detergent‐effect.

It is clear the impact of Aβ on the lipid membrane is dependent on the specific assembly forms of Aβ structures. Oligomers, curvilinear protofibrils and annular structures are responsible for membrane disruption. The flexible dynamic structures with exposed hydrophobic residues are capable of interacting and inserting into the membrane more readily than Aβ monomers or fibrils. There appears to be two distinct actions which these Aβ assemblies can exert directly on the lipid bilayer, Figure 10.

Firstly, Aβ_42_ but not Aβ_40_ oligomers, can insert into the bilayer and promote large, non‐selective, ion‐channel pores.^[22]^ The size of conductance suggests an internal diameter of the pore of 1.9; 2.1 and 2.4 nm.^[22]^ This matches the internal diameter reported for barrel shaped annular oligomers, imaged by TEM^[97b]^ and AFM,[^32^, ^59^] Figure 10A. Secondly, Aβ_42_ but also Aβ_40_ oligomers, together with the more extended curvilinear protofibrils will insert and carpet the upper leaflet of the lipid bilayer.^[23]^ The effect is widespread and causes a permeability to ions, in particular Ca^2+^.[^13d^, ^59^, ^69a^, ^119a^, ^121^, ^122^] This Aβ insertion may also cause some extraction of lipid from the bilayer, described as a detergent‐effect, Figure 10B. Aβ oligomers appear to migrate and concentrate at the interface between two lipid membranes.^[23]^ This suggests clustering of Aβ curvilinear‐protofibrils might occur at synaptic junctions and so disrupt synaptic activity.^[8]^

These effects imaged by cryoET, AFM and TEM match the cytotoxicity studies and permeability measurements that indicate Aβ prefibrillar oligomers together with extended‐ curvilinear and annular protofibrils are the most cytotoxic assemblies of Aβ.[^13c^, ^13d^, ^13f^] The ‘carpeting‐effect’ imaged for Aβ oligomers seem to be equally marked for both Aβ_40_ or Aβ_42_ isoforms. However, AD pathology and cytotoxicity point to Aβ_42_ as being more toxic.[^13f^, ^15^, ^16^] This may be because in vivo levels of Aβ_42_ oligomers exceed that of Aβ_40_.

An interesting observation indicates Aβ oligomers produced via secondary‐nucleation appear to be cytotoxic, while oligomers permitted to form only through primary‐nucleation are not.[^84^, ^145^] This suggests that oligomers formed via secondary‐nucleation are either structurally distinct from oligomers produced via primary nucleation, or the quantity of oligomers produced on the pathway to fibrils is much greater for a secondary‐nucleation pathway. In terms of therapeutic approaches this is an exciting prospect as it indicates that conversion of Aβ monomers to fibrils by primary‐nucleation can occur without cytotoxicity.[^84^, ^145^] Inhibition of just secondary‐nucleation, maybe an effective therapeutic approach.[^37^, ^84^, ^145a^, ^146^] In support of this approach, Aducanumab, which has been approved by the USA Drug agency as an AD therapy, has been shown to inhibit secondary nucleation.^[145a]^ In addition, molecules that block Aβ ion channels, inhibit their formation or insertion into the membrane, represent a promising therapeutic approach.[^93^, ^103^, ^147^]

This review has largely focused on Aβ from Alzheimer's and its impact on membranes and cytotoxicity, but many of the observations described in this review draw parallels with the impact of other amyloid protein oligomers on the membrane.[^32^, ^33^] Examples include: α‐Synuclein from Parkinson's disease;[^30^, ^148^] Amylin, or islet amyloid polypeptide (*IAPP*) for diabetes;[^31^, ^149^] β_2_macroglobulin (β_2_M);^[76]^ serum amyloid‐A^[77]^ and mammalian prion protein.^[150]^ Furthermore, many of the impacts Aβ has on the membrane are similar to the action of anti‐microbial peptides.[^17b^, ^34^, ^35^, ^36^, ^151^]

Although much has been discovered about Aβ‐membrane interactions, much remains to be understood. High resolution cryoEM structures of annular oligomers that will inform rational drug design are yet to be determined. Furthermore, the precise structural relationship between small Aβ oligomers, curvilinear protofibrils, annular structures and fibrils is not resolved. Nanoscale imaging of Aβ interaction with cellular membranes ex vivo is now an exciting prospect. The level and form of Aβ oligomers sufficient to overwhelm the cell with Ca^2+^ influx needs to be determined. Finally, it is yet to be established if Aβ cytotoxicity action in vivo is independent of cell surface receptors, whether these effects dominate cytotoxicity or are only players in this devastating multi‐factorial disease.

## Conflict of interest

The authors declare no conflict of interest.

## Biographical Information

*Dr John H. Viles uses a range of biophysical techniques to study the fundamental processes that involve neurotoxic oligomer and amyloid fibril formation in Alzheimer's, Parkinson's and Prion disease. Approaches include biomolecular spectroscopies (including NMR and CD) together with molecular resolution microscopy (cryo‐Electron and Atomic Force Microscopy). He received his Ph.D. from the University of London in 1994 and in 1997 he took up a post‐doctoral position at the Scripps Research Institute, California to study the structure of the prion protein. He returned to the UK in 2000 to take up a lectureship position and is currently an associate professor in biochemistry at Queen Mary, University of London*.